# Return to Sport After Subtalar Arthroereisis in Pediatric Flexible Flatfoot: Radiographic Correction and Residual Pain Are Not Independent Predictors

**DOI:** 10.3390/children13050632

**Published:** 2026-05-01

**Authors:** Sergio De Salvatore, Costanzo Testa, Silvia Salera, Amos Cocola, Leonardo Oggiano, Edoardo Costici, Fabrizio Donati, Laura Ruzzini, Fabio Pascarella, Paolo Brigato, Pier Francesco Costici

**Affiliations:** 1 Orthopedic and Traumatology Unit, Department of Surgery, Bambino Gesù Children’s Hospital, 00165 Rome, Italy; leonardo.oggiano@opbg.net (L.O.); fabrizio.donati@opbg.net (F.D.); laura.ruzzini@opbg.net (L.R.); pierfrancesco.costici@opbg.net (P.F.C.); 2 Orthopaedic and Traumatology Department, Policlinico Universitario Agostino Gemelli, 00136 Rome, Italy; costatesta77@gmail.com (C.T.); fabio.pascarella@opbg.net (F.P.); 3 Orthopaedic and Traumatology Department, Policlinico Universitario Tor Vergata, 00133 Rome, Italy; silvia.salera.24@gmail.com; 4 Research Unit of Orthopaedic and Trauma Surgery, Department of Medicine and Surgery, Università Campus Bio-Medico di Roma, Via Alvaro del Portillo 21, 00128 Roma, Italy; amoscosimo.cocola@unicampus.it; 5 Faculty of Medicine and Surgery, Saint Camillus International University of Health Sciences, 00131 Roma, Italy; edoardo.costici@uniroma1.it

**Keywords:** subtalar arthroereisis, pediatric flexible flatfoot, return to sport, radiographic correction, UCLA activity score, sinus tarsi pain

## Abstract

**Highlights:**

**What are the main findings?**

- Subtalar arthroereisis achieved consistent radiographic correction, but neither correction magnitude nor residual sinus tarsi pain was associated with return to sport.
- Early postoperative activity level (UCLA score at 3 months) was the only independent predictor of return to sport.

**What are the implications of the main findings?**

- Return to sport after arthroereisis depends more on early functional recovery than on radiographic alignment or pain alone.
- Postoperative evaluation should integrate functional assessment and rehabilitation strategies rather than relying solely on imaging correction.

**Abstract:**

**Background/Objectives:** Subtalar arthroereisis (STA) is widely used for symptomatic pediatric flexible flatfoot and provides consistent radiographic correction. However, return to sport (RTS) after STA is less well defined, and the relative role of early postoperative pain versus radiographic correction remains unclear. **Methods**: We performed a retrospective observational cohort study of consecutive skeletally immature patients treated with STA using a non-absorbable endosinotarsal screw at a single tertiary center. Inclusion criteria were symptomatic flexible flatfoot refractory to >6 months of conservative treatment, complete pre-/postoperative weight-bearing radiographs, complete functional data, and minimum follow-up of 6 months. The primary endpoint was RTS to the pre-symptom primary sport at final follow-up. Secondary outcomes were UCLA Activity Score, FAAM Sport subscale, and postoperative VAS. Radiographic correction was quantified as delta change (Δ) in Meary, Costa–Bertani, and Kite angles. Group comparisons used nonparametric tests. A parsimonious multivariable logistic regression model (EPV-constrained) included UCLA at 3 months, VAS pain, and ΔMeary. **Results**: Fifty patients were included (mean follow-up 9.2 ± 2.5 months; range 6–14); 27/50 (54%) resumed their primary sport. Baseline characteristics were comparable between Returners and Non-Returners. Returners showed higher early postoperative UCLA scores than Non-Returners (8.0 [7.0–8.5] vs. 6.0 [5.0–7.0], *p* = 0.005). FAAM Sport and VAS pain did not differ significantly between groups (*p* = 0.224 and *p* = 0.493, respectively). Radiographic correction magnitude was similar between groups (ΔMeary *p* = 0.938; ΔCosta–Bertani *p* = 0.984; ΔKite *p* = 0.108). In multivariable analysis, UCLA at 3 months was the only independent correlate of RTS (OR 2.65 per point, 95% CI 1.34–6.15; *p* = 0.009), whereas VAS pain (OR 0.98, 95% CI 0.76–1.25; *p* = 0.892) and ΔMeary (OR 1.01, 95% CI 0.91–1.13; *p* = 0.875) were not significant. **Conclusions**: In this cohort, STA achieved substantial radiographic correction, but neither correction magnitude nor early postoperative pain independently correlated with RTS at short-term follow-up. Early postoperative activity level was the strongest independent correlate of sport resumption, supporting a function-centered postoperative assessment beyond radiographic alignment alone.

## 1. Introduction

Symptomatic flexible flatfoot (planovalgus deformity) is a frequent cause of referral in pediatric orthopedics. Although most cases are managed conservatively, surgical treatment may be indicated when deformity persists and is associated with activity-limiting pain or fatigue [1,2]. Among surgical options, subtalar arthroereisis (STA) with an endosinotarsal screw has been increasingly adopted because of its minimally invasive profile and its biomechanical effect in limiting excessive subtalar eversion, thereby supporting restoration of the medial longitudinal arch [3,4]. Recent prospective adolescent data have further supported improvements in clinical and radiographic outcomes after STA for symptomatic flexible flatfoot [5]. At the health-system level, nationwide analyses have reported a growing volume of pediatric flatfoot surgery in recent years, further underscoring the clinical importance of robust outcome assessment after STA [6].

The radiographic efficacy of STA has been consistently reported. Several medium- and long-term studies have shown improvement in static geometric parameters, including Meary’s angle and calcaneal pitch, with a trend toward restoration of more physiological alignment [7,8]. However, definitions of surgical success have remained predominantly anatomically centered, relying mainly on radiographic correction and less frequently addressing dynamic, patient-relevant outcomes such as RTS, sports performance, quality of life, and patient-perceived recovery [9,10]. In pediatric and adolescent patients, resumption of sports participation is often a major expectation for both patients and families. Nevertheless, evidence on RTS rates and barriers after arthroereisis remains limited. In addition, STA is not free of complications; residual sinus tarsi pain is the most commonly reported adverse event, with prevalence estimates ranging from 4% to 18%, and represents a frequent indication for implant removal [11,12,13].

This context raises an important clinical issue: persistent postoperative pain may not necessarily limit sports reintegration, as return to sport may also be influenced by non-structural and behavioral factors, including psychological readiness and kinesiophobia, similar to observations in other orthopedic settings such as ACL reconstruction [14]. To date, few studies have directly examined the relationship between radiographic correction magnitude, residual pain intensity, and RTS in this specific population.

Accordingly, this study aimed to evaluate RTS in a cohort of pediatric patients treated with STA and to assess whether radiographic correction magnitude and residual pain were independently associated with sports resumption. We hypothesized that complete radiographic normalization is not mandatory for functional recovery, and that RTS is more closely related to overall activity capacity than to pain absence alone.

## 2. Materials and Methods

### 2.1. Study Design and Reporting Standards

This retrospective observational cohort study included a consecutive series of pediatric patients treated for symptomatic flexible flatfoot at a single tertiary institution. The study was designed, conducted, and reported in accordance with the STROBE (Strengthening the Reporting of Observational Studies in Epidemiology) recommendations for cohort studies. The study was conducted in accordance with the Declaration of Helsinki. Given the retrospective design and the use of fully anonymized data collected during routine clinical practice, formal Institutional Review Board approval and individual informed consent were waived according to local institutional policy and applicable regulations.

### 2.2. Setting

All patients were managed and followed at the same institution through a dedicated pediatric foot and ankle pathway. Surgical indication, perioperative care, radiographic follow-up, and functional evaluation were applied using a standardized institutional protocol.

### 2.3. Patient Selection (STROBE Item 6)

We screened all skeletally immature patients who underwent subtalar arthroereisis (STA) with a non-absorbable endosinotarsal screw during the study period.

Inclusion criteria were as follows:

1. Symptomatic flexible flatfoot refractory to conservative treatment (orthoses and physiotherapy for >6 months);
2. Complete preoperative and postoperative weight-bearing radiographic datasets;
3. Complete functional outcome data at final follow-up;
4. Minimum clinical follow-up of 6 months.

Exclusion criteria were as follows:

1. Rigid flatfoot (including tarsal coalition);
2. Neurological or neuromuscular disorders affecting gait/function;
3. Previous foot/ankle surgery;
4. Incomplete questionnaires or missing key outcome data.

All included patients had follow-up beyond 6 months.

### 2.4. Unit of Analysis and Bilateral Cases

The unit of analysis was the patient (not the foot). RTS status was defined at patient level. In bilateral procedures, radiographic correction metrics were derived from the prespecified index side (most symptomatic side at baseline; if symptoms were symmetrical, right side by convention) to preserve statistical independence.

### 2.5. Surgical Technique

All procedures were performed by one senior surgeon using a standardized technique. Under fluoroscopic guidance, the sinus tarsi was approached by blunt dissection. Trial sizing was performed to select the implant that allowed physiological eversion (approximately 2–4°) without overcorrection, followed by insertion of the definitive non-absorbable endosinotarsal screw. No adjunctive tendon procedures (e.g., Achilles lengthening) were performed in this cohort. No postoperative immobilization with casts or braces was required. After unilateral procedures, patients were allowed to ambulate with weight-bearing as tolerated from postoperative day 4 using two crutches; in bilateral procedures, weight-bearing as tolerated was allowed from postoperative day 7. Crutches were generally discontinued after 14 days. Swimming was permitted after 1 month, whereas other sports activities were generally allowed after 3 months according to clinical progression. Physiotherapy was not routinely prescribed and was performed only in patients who were still unable to walk adequately after the first 14 postoperative days.

### 2.6. Data Collection and Timepoints

Outcomes were collected at two predefined timepoints:

- Baseline (T0): preoperative assessment including age, sex, anthropometrics (if available), primary sport participation, and standard weight-bearing radiographs.
- Final Follow-Up (T1): most recent clinical evaluation available, with follow-up duration ≥ 6 months for all included patients.

At T1, patients were classified as follows:

- Returners: they resumed their pre-symptom primary sport. Return to sport was defined as resumption of the pre-symptom primary sport at the same level of participation, as reported by the parents in the presence of the patient during outpatient clinical follow-up. Owing to the retrospective design, training frequency and intensity were not recorded as separate variables, and no external objective verification was available.
- Non-Returners: they did not resume their pre-symptom primary sport.

Where available from records, time from surgery to first RTS was documented. The follow-up window adopted in the present study was intended to capture early postoperative recovery and initial return-to-sport behavior, which represented the primary clinical focus of the analysis, rather than long-term maintenance of sports participation.

### 2.7. Outcomes

The primary clinical endpoint was return to sport (RTS) at final follow-up (binary, patient-level).

Secondary outcomes included the following:

1. UCLA Activity Score (1–10), to quantify global activity level;
2. FAAM Sport subscale (0–100), to assess sport-related function;
3. VAS pain score (0–10), defined as postoperative pain intensity measured at the first postoperative clinical follow-up. This variable was collected as a general postoperative pain measure and did not distinguish between resting pain and activity-related pain.

Radiographic correction was quantified as delta change (Δ) between T0 and T1 on weight-bearing radiographs for

- Meary’s angle (sagittal talo–first metatarsal alignment);
- Costa–Bertani angle (medial longitudinal arch profile);
- Kite’s angle (hindfoot alignment).

For each parameter, direction of correction was handled consistently so that Δ represented the magnitude of postoperative correction.

For prognostic modeling, the UCLA Activity Score used in multivariable analysis was the value recorded at the 3-month postoperative intermediate visit, to represent early recovery aptitude rather than final-status activity.

### 2.8. Bias Control, Measurement Quality and Missing Data

To reduce detection bias, radiographic measurements were performed digitally by two independent assessors blinded to clinical outcomes and RTS status. Inter-observer reliability was assessed using intraclass correlation coefficients (ICCs), yielding values of 0.88 for Meary angle, 0.84 for Costa–Bertani angle, and 0.81 for Kite angle. Intra-observer reliability was similarly good to excellent, with ICC values of 0.91, 0.87, and 0.85, respectively. Missingness was quantified per variable. Primary analyses used a complete-case approach with explicit denominators for each analysis. No data-driven imputation was performed.

### 2.9. Statistical Analysis

Statistical analyses were performed using R software (R Foundation for Statistical Computing, Vienna, Austria; version 2026.01.0+392 (2026.01.0+392)). Distributional assumptions were assessed with the Shapiro–Wilk test. Continuous variables were summarized as median and interquartile range (IQR) [range] and compared between groups with the Mann–Whitney U test. Categorical variables were reported as counts (percentages) and compared using Fisher’s exact test. Bivariate associations were explored with Spearman’s rank correlation coefficient (ρ).

Given the sample size (n = 50) and number of RTS events (n = 27), the multivariable analysis was prespecified as parsimonious to reduce overfitting risk. The final logistic regression model was restricted to three covariates (Events-Per-Variable ratio = 9.0): (1) UCLA Activity Score at the 3-month postoperative visit (early functional recovery marker), (2) postoperative pain at the first postoperative clinical follow-up (VAS), and (3) radiographic correction magnitude (ΔMeary angle). Continuous predictors were modeled on their native scale (no data-driven dichotomization). Effect estimates were reported as odds ratios (ORs), 95% confidence intervals (95% CIs), regression coefficients (β), standard errors (SE), and exact two-sided *p*-values.

Model diagnostics included multicollinearity assessment (variance inflation factor, VIF) and calibration assessment (Hosmer–Lemeshow test). A two-sided *p*-value < 0.05 was considered statistically significant. Because of the exploratory nature of multivariable modeling in a modest cohort, interpretation prioritized effect-size precision (95% CIs) rather than *p*-values alone. Additional exploratory post hoc analyses were performed to assess the potential association between achievement of normative radiographic thresholds and return to sport.

## 3. Results

### 3.1. Study Population and Baseline Characteristics

A total of 198 feet (105 patients) treated with subtalar arthroereisis during the study period were screened for eligibility. Fifty-five patients were excluded because of incomplete functional survey data or loss to follow-up and were retained only in the radiographic database. Four additional patients were excluded due to follow-up <6 months. The final cohort therefore comprised 50 patients. Because all procedures were bilateral, 100 feet were treated; however, the unit of analysis was the patient, and radiographic analyses were performed on the prespecified index side. The mean follow-up was 9.2 ± 2.5 months (range, 6–14 months). Exact time-to-RTS data were available for 38/50 patients; in the remaining cases, RTS was recorded as binary status (Return/Non-Return) at final follow-up interview.

At final follow-up, 27 patients (54%) had resumed their pre-symptom primary sport (Returners), whereas 23 (46%) had not (Non-Returners). Baseline characteristics were comparable between groups (Table 1), with no significant differences in age at surgery (*p* = 0.286), sex distribution (*p* = 0.771), laterality (*p* = 1.000), or proportion of contact sports (*p* = 0.573).

### 3.2. Clinical Outcomes and Radiographic Correction by RTS Status

**Figure 1:**
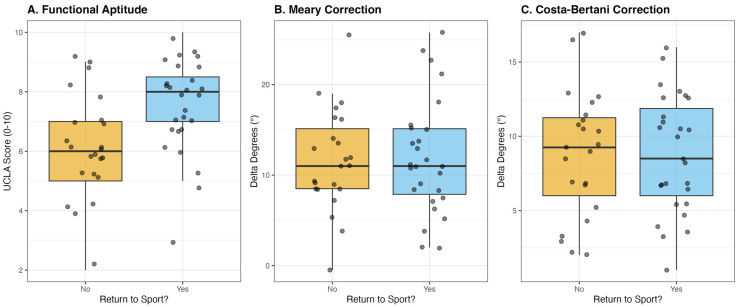
Boxplots of Functional Activity and Radiographic Correction by Return-to-Sport Status.

Inter- and intra-observer reliability for radiographic measurements was good to excellent (inter-observer ICC range, 0.81–0.88; intra-observer ICC range, 0.85–0.91). Early postoperative global activity level differed between groups. Returners showed significantly higher UCLA Activity Scores than Non-Returners (8.0 [7.0–8.5] vs. 6.0 [5.0–7.0], *p* = 0.005). In contrast, FAAM Sport scores were similar (70.0 [50.0–77.5] vs. 65.0 [47.5–75.0], *p* = 0.224), and early postoperative pain (VAS) measured at the first postoperative clinical follow-up did not differ significantly (6.0 [0.0–6.5] vs. 6.0 [3.5–7.0], *p* = 0.493) (Table 2, Figure 1). Importantly, this measure reflects pain at the early postoperative follow-up rather than pain at the time of final return-to-sport assessment.

**Figure 2:**
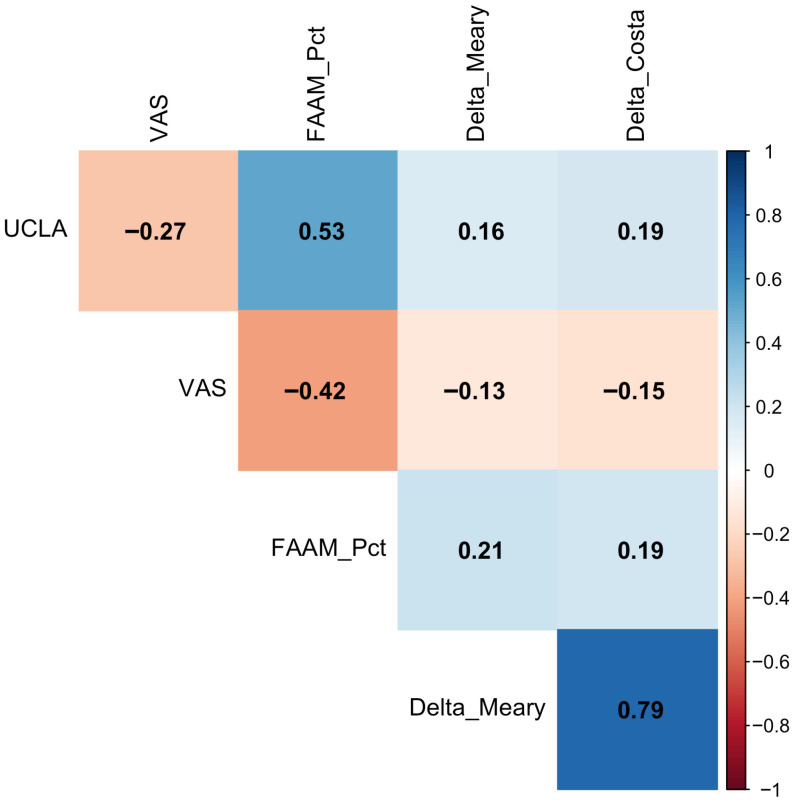
Correlation analysis.

STA produced substantial radiographic correction in the overall cohort. However, correction magnitude was not associated with RTS status. No significant between-group differences were observed for ΔMeary angle (11.0 [7.9–15.1] vs. 11.0 [8.5–15.1], *p* = 0.938), ΔCosta–Bertani angle (8.5 [6.0–11.9] vs. 9.2 [6.0–11.2], *p* = 0.984), or ΔKite angle (3.8 [1.0–6.9] vs. 5.8 [3.1–9.2], *p* = 0.108) (Table 2). In an exploratory post hoc analysis, achievement of a postoperative Meary angle < 15° was not associated with return-to-sport status, with comparable RTS rates observed between patients reaching this threshold and those with residual deformity. Correlation analysis showed near-null associations between radiographic correction magnitude and functional outcomes (Figure 2).

### 3.3. Multivariable Predictors of Return to Sport

A prespecified parsimonious multivariable logistic regression model was fitted to assess independent correlates of RTS. Given cohort size (n = 50) and number of events (n = 27), model complexity was restricted to three covariates (EPV = 9.0): UCLA Activity Score at the 3-month postoperative visit, VAS postoperative pain at the first postoperative clinical follow-up, and ΔMeary angle.

In this model, UCLA Activity Score at 3 months was the only significant independent correlate of RTS (OR 2.65 per 1-point increase, 95% CI 1.34–6.15; *p* = 0.009). VAS postoperative pain (OR 0.98, 95% CI 0.76–1.25; *p* = 0.892) and ΔMeary correction (OR 1.01, 95% CI 0.91–1.13; *p* = 0.875) were not independently associated with RTS (Table 3). Diagnostic checks showed acceptable calibration (Hosmer–Lemeshow *p* > 0.05) and no relevant multicollinearity (all VIFs < 1.5).

## 4. Discussion

In this retrospective cohort of pediatric patients with symptomatic flexible flatfoot treated with subtalar arthroereisis, three main findings emerged. First, return to sport (RTS) to the pre-symptom primary sport was achieved in 54% of patients at short-term follow-up. This RTS rate appears lower than that reported in previous studies, where rates exceeding 90% have been described, including recent large mid-term series of calcaneo-stop arthroereisis reporting high functional outcomes and high rates of sports resumption [15]. This difference is likely explained by methodological factors. In the present study, RTS was defined stringently as return to the pre-symptom primary sport at the same level of participation, whereas prior studies have often adopted broader definitions of postoperative sports resumption. In addition, our mean follow-up was 9.2 months, so some patients classified as Non-Returners may represent delayed Returners rather than definitive failures. Second, although radiographic correction was substantial overall, the magnitude of correction was not associated with RTS status. Third, residual sinus tarsi pain did not independently discriminate Returners from Non-Returners, whereas early postoperative global activity level (UCLA at 3 months) was the only independent correlate of RTS. Although longer follow-up is necessary to evaluate the durability of sports participation over time, the follow-up interval used in the present study was intentionally centered on the early postoperative phase, when return-to-sport decisions are most clinically relevant for patients, families, and surgeons. Accordingly, the present findings should be interpreted as referring to early postoperative return-to-sport behavior rather than to the long-term durability of athletic participation or the risk of late complications, which have been addressed more directly in recent long-term series of pediatric subtalar arthroereisis with metallic implants [16,17].

These findings support a function-centered interpretation of recovery after STA. In our cohort, patients with comparable angular correction (ΔMeary, ΔCosta–Bertani, and ΔKite) showed different sport trajectories, indicating that structural realignment alone does not explain sport reintegration. Consistently, an exploratory post hoc analysis based on achievement of a postoperative Meary angle < 15° did not show a significant difference in return-to-sport rate compared with patients with residual deformity. Although this threshold-based comparison should be interpreted cautiously because of the small number of patients with persistent radiographic abnormality, it further supports the notion that radiographic alignment alone may not fully explain sport reintegration. This observation is consistent with the broader literature showing that STA can improve alignment reliably, while functional outcomes are influenced by multiple interacting domains beyond static radiographic metrics [18]. Recent systematic evidence on calcaneo-stop arthroereisis further supports the overall effectiveness of this procedure in symptomatic pediatric flexible flatfoot, while also highlighting the heterogeneity of outcome definitions across studies.

A clinically relevant result was the dissociation between pain intensity and sport behavior. This finding should not be interpreted as evidence that patients resumed sport despite high pain levels at the time of RTS. Rather, it indicates that pain intensity recorded at the first postoperative follow-up did not discriminate between those who later resumed sport and those who did not. Psychological factors, including kinesiophobia, reduced confidence with loading, and limited psychological readiness, may also have contributed to this dissociation, although these variables were not directly measured.

The relatively high proportion of Non-Returners should be interpreted cautiously, as non-return to sport is unlikely to reflect a single mechanism. In addition to residual symptoms, it may also be influenced by fear of reinjury, reduced confidence with activity, family or clinician caution, lower motivation to resume the pre-symptom sport, or changes in sport preference during follow-up.

In contrast, early postoperative activity profile was strongly associated with later RTS. The early UCLA score should be interpreted as an integrated marker of recovery rather than as an isolated mechanistic determinant. It may reflect potentially modifiable factors such as rehabilitation progression and confidence with activity but also unmeasured patient-level characteristics including baseline activity, motivation, family support, and psychological readiness. Therefore, although each 1-point increase in UCLA score at the 3-month visit was associated with a marked increase in the odds of RTS, the present data do not allow identification of which components most strongly influenced this association.

From an implementation perspective, these data suggest that postoperative assessment after STA should combine radiographic monitoring with standardized functional profiling, rather than using angular correction as a surrogate of sport recovery. Prior RTS-focused evidence in pediatric STA remains limited [19]; therefore, incorporating explicit return-to-sport milestones into follow-up pathways may improve counseling of patients and families and better align surgical success metrics with patient priorities.

Overall, our results reinforce the idea that radiographic correction is important for deformity treatment but may be insufficient to predict sport reintegration at the individual level [20]. A multidimensional recovery model appears more appropriate for RTS-oriented decision-making after pediatric STA.

### Limitations

This study has several limitations inherent to its retrospective design. Although patients were included consecutively, the single-center setting may limit external generalizability across institutions with different indications, rehabilitation pathways, and sport counseling practices. The sample size was modest (n = 50), and with 27 RTS events, we constrained multivariable modeling to three covariates (EPV = 9.0) to reduce overfitting risk. A major limitation is the substantial reduction in the initial sample, as a large proportion of screened patients were excluded mainly because of incomplete functional data or loss to follow-up. Because this missingness may not have been random, selection bias cannot be excluded, and the reported RTS rate may either overestimate or underestimate the true rate in the overall treated population. In addition, variables such as BMI, rehabilitation adherence, sport exposure, socioeconomic context, implant position, gait parameters, and pedobarographic data were not systematically available and could not be included in the analysis; therefore, residual confounding cannot be excluded. Time-to-RTS data were not available for the entire cohort, so the primary endpoint was binary RTS status at final follow-up, which reduced temporal granularity and may have misclassified delayed Returners. The follow-up duration (minimum 6 months; mean 9.2 months) is appropriate for early postoperative assessment but does not capture long-term durability of sport participation or late implant-related outcomes. Outcome phenotyping was also limited: RTS was defined as return to the pre-symptom primary sport at the same reported participation level, without formal quantification of competition level, training volume, or sustained participation over time. Finally, physiotherapy was delivered only on a needs basis rather than through a standardized rehabilitation program, which may have influenced early functional recovery, UCLA scores, and return-to-sport timing

## 5. Conclusions

In this cohort of pediatric patients undergoing subtalar arthroereisis for symptomatic flexible flatfoot, substantial radiographic correction was achieved, but correction magnitude was not independently associated with return to sport at short-term follow-up. Residual sinus tarsi pain was also not an independent correlate of RTS. Early postoperative global activity level (UCLA at 3 months) was the strongest independent correlate of sport resumption. These findings support a function-centered postoperative framework in which radiographic assessment is integrated with early functional stratification to guide rehabilitation and counseling. Prospective multicenter studies with longer follow-up and more granular RTS phenotyping are warranted to validate and refine these observations. These findings support a function-centered postoperative framework in which radiographic assessment is integrated with early functional stratification to guide rehabilitation and counseling; however, they should be interpreted within the context of short-term follow-up. Prospective multicenter studies with longer follow-up and more granular RTS phenotyping are warranted to validate and refine these observations

## Figures and Tables

**Table 1:** Demographics.

Characteristics	Non-Returners	Returners	*p*-Value
Patients, n	23	27	-
Age at surgery (years)	11.8 (10.5–13.0) [9.0–15.0]	11.3 (10.2–12.5) [9.0–14.0]	0.286
Male sex, n (%)	15 (65.2%)	16 (59.3%)	0.771
Laterality (bilateral)	23 (100%)	27 (100%)	1.000
Contact Sport (yes)	13 (56.5%)	13 (48.1%)	0.573

Data are presented as median (IQR) [range] for continuous variables and n (%) for categorical variables.

**Table 2:** Outcome Comparison.

Outcome	Non-Returners	Returners	*p*-Value
UCLA Activity Score	6.0 (5.0–7.0) [2.0–9.0]	8.0 (7.0–8.5) [3.0–10.0]	0.005 *
FAAM Sport Score	65.0 (47.5–75.0) [20.0–100.0]	70.0 (50.0–77.5) [25.0–100.0]	0.224
VAS Pain Score	6.0 (3.5–7.0) [0.0–9.0]	6.0 (0.0–6.5) [0.0–8.0]	0.493
Delta Meary (Correction °)	11.0 (8.5–15.1) [−0.5–25.5]	11.0 (7.9–15.1) [2.0–25.8]	0.938
Delta Costa–Bertani (Correction °)	9.2 (6.0–11.2) [2.0–17.0]	8.5 (6.0–11.9) [1.0–16.0]	0.984
Delta Kite (Correction °)	5.8 (3.1–9.2) [0.0–14.5]	3.8 (1.0–6.9) [−3.2–17.5]	0.108

Values: Median (IQR) [Range]. *p*-values: Mann–Whitney U Test. bold mean *p* < 0.05; * ≤ 0.05

**Table 3:** Logistic Regression.

Covariate	Odds Ratio (OR)	95% Confidence Interval (CI)	Coefficient (β)	Standard Error (SE)	*p*-Value
UCLA Activity Score (3-month postoperative)	2.65	1.34–6.15	0.97	0.35	0.009 *
VAS postoperative pain	0.98	0.76–1.25	−0.02	0.12	0.892
ΔMeary angle (°)	1.01	0.91–1.13	0.01	0.05	0.875

Outcome coded as Returner vs. Non-Returner. Final parsimonious model included UCLA Activity Score (3-month postoperative), VAS postoperative pain, and ΔMeary angle. EPV = 9.0. OR indicates change in odds per 1-unit increase in predictor. *p*-values are two-sided. * ≤ 0.05.

## Data Availability

The datasets used and/or analyzed during the current study are not publicly available due to our policy statement of sharing clinical data only on request but are available from the corresponding authors on reasonable request.

## Abbreviations

β	Beta coefficient
CI	Confidence Interval
EPV	Events Per Variable
FAAM	Foot and Ankle Ability Measure
ICC	Intraclass Correlation Coefficient
IQR	Interquartile Range
OR	Odds Ratio
RTS	Return to Sport
SE	Standard Error
STA	Subtalar Arthroereisis
STROBE	Strengthening the Reporting of Observational Studies in Epidemiology
T0	Baseline
T1	Final Follow-up
UCLA	University of California, Los Angeles Activity Score
VAS	Visual Analog Scale
VIF	Variance Inflation Factor
Δ	Delta change
ρ	Spearman’s rank correlation coefficient
